# Using Extinction-Renewal to Circumvent the Memory Strength Boundary Condition in Fear Memory Reconsolidation

**DOI:** 10.3390/brainsci11081023

**Published:** 2021-07-31

**Authors:** Tiffany L. Campbell, Daniel E. Kochli, Mitch A. McDaniel, Mallory K. Myers, Mallory E. Dunn, Victoria A. Diana, Jennifer J. Quinn

**Affiliations:** 1 Department of Psychology and Center for Neuroscience & Behavior, Miami University, 90 N. Patterson Ave., Oxford, OH 45056, USA; campbe73@miamioh.edu (T.L.C.); mcdanim5@miamioh.edu (M.A.M.); myersmk@miamioh.edu (M.K.M.); dunnme2@miamioh.edu (M.E.D.); dianava@miamioh.edu (V.A.D.); 2 Department of Psychology, Washington College, 300 Washington Avenue, Chestertown, MD 21620, USA; dkochli2@washcoll.edu

**Keywords:** destabilization, midazolam, overtraining, learning, rat

## Abstract

Reconsolidation is a process by which memories are destabilized, updated, and then restabilized. Strong memories are resistant to undergoing reconsolidation. Here, we addressed whether an overtrained fear memory could be made susceptible to reconsolidation by first extinguishing, and then renewing, the memory. Rats were trained with ten tone-footshock pairings, followed by eight days of tone extinction in the training context. The next day, rats were placed into a second context and memory for the tone was renewed/reactivated with a single tone presentation. Immediately following reactivation, rats received an injection of midazolam or vehicle. Rats were then tested for freezing to the tone in a third context. Midazolam had no effect in rats that did not undergo tone extinction, but significantly attenuated freezing to the tone in extinguished rats. Thus, rats that received tone extinction underwent tone memory reconsolidation following its renewal. In a second experiment, we administered the reactivation session and midazolam injections prior to extinction. Midazolam had no effect and rats extinguished at a rate similar to controls. These data suggest that strong emotional memories are capable of updating following weakening of memory expression through extinction.

## 1. Introduction

Reconsolidation is a protein synthesis-dependent process by which long-term, stabilized memories may be updated in order to incorporate new information [1,2,3]. Upon retrieval, the stabilized memory may enter into a labile state where it becomes susceptible to disruption or enhancement via behavioral and/or pharmacological manipulation [4,5,6,7,8]. Often, the occurrence of memory reconsolidation is demonstrated by a lasting disruption of the memory following protein synthesis inhibition at the time of retrieval, e.g., [5]. In addition to protein synthesis inhibition, administrations of other pharmacological agents have similarly been shown to block memory reconsolidation. Midazolam (MDZ), a GABA-A receptor agonist, disrupts memory reconsolidation in fear conditioning [9,10,11]; but see [12] and morphine conditioned place preference [13] paradigms.

Recently, a number of investigations have revealed that retrieval alone is sometimes not sufficient to initiate reconsolidation processes. As such, specific “boundary conditions” have been identified that determines whether a memory undergoes reconsolidation [10,14,15,16]. One such boundary condition is the strength of the memory, with the general consensus being that stronger memories are more resistant to reconsolidation [13,14,15,17,18,19]. Unfortunately, this presents a challenge for the translation of reconsolidation-based interventions to psychiatric disorders given that these disorders often involve particularly strong memories, such as those that underlie posttraumatic stress disorder and substance use disorders [19,20].

Efforts to circumvent these boundary conditions are increasing in the literature. For example, a number of studies have been successful in disrupting reconsolidation-resistant memories by extending the duration of the reactivation sessions/trials [10,14,21], increasing the lability of a resistant memory using pharmacological agents [22,23], using an unexpected retrieval or adding a novel component to reactivation sessions thus increasing the prediction error [11,24], or increasing the dose of the amnestic agent used to block reconsolidation [10]. Given these recent successes, it is encouraging to think of these boundary conditions as challenging, rather than absolute [19].

In the present experiments, we addressed whether the resistance to reconsolidation that is observed with strong fear memories could be overcome by first reducing the behavioral expression of the memory. We hypothesized that extinction of a strong fear memory followed by partial renewal of that memory in conjunction with midazolam would yield disruption of reconsolidation of the fear memory. Repeated presentation of a previously reinforced stimulus in the absence of reinforcement yields extinction of responding, but not forgetting or erasure of the original memory [25,26]. However, subsequent presentation of that stimulus in another context allows for renewal of the responding, albeit usually at a lower level than that observed at the end of acquisition [26,27]. Thus, renewed fear responding at sub-asymptotic levels may allow the formerly resistant strong memory to undergo reconsolidation.

## 2. Materials and Methods

### 2.1. Animals

Seventy-six experimentally naïve adult male Long-Evans rats (Harlan Laboratories, Indianapolis, IN, USA) were used in this experiment. Rats were pair-housed at 23 °C and 50% humidity on a 14:10 h light:dark cycle with ad libitum access to food and water. Rats were handled by each experimenter for approximately 30 s per day for five consecutive days prior to experimentation. All procedures were performed during the light cycle and were approved by the Miami University Institutional Care and Use Committee in accordance with the NIH Guidelines for the Care and Use of Experimental Animals.

### 2.2. Behavioral Apparatus

Animals underwent experimentation in three distinct contexts. Each context consisted of four identical conditioning chambers (32.4 × 25.4 × 21.6 cm^3^; Med-Associates, Inc., Georgia, CT, USA) housed within sound-attenuating cubicles. Chambers were composed of a white plastic back wall, aluminum side walls, and a transparent Plexiglas ceiling and front door. Rats were continuously monitored using progressive scan video cameras (VID-CAM-MONO-4; Med Associates, Inc., Fairfax, VT, USA) connected to a computer operating Video Freeze software (Med Associates, Inc.) to automatically detect defensive freezing behavior. Freezing behavior is defined as the cessation of all movements not required for respiration, e.g., [28].

Context A chambers were brightly lit (125 lux) and contained a flat grid floor composed of 19 equally spaced stainless-steel rods. Pans underlying the grid floors contained approximately 10 mL of 50% vanilla extract solution (Kroger). Grid floors were wired to a shock generator and scrambler (Med-Associates, Inc.). Odorless sodium hydroxide (5%) was used to clean chambers before each rat was placed inside. Context B chambers were located in a distinctly different room than Context A and lighted with near-infrared light (0 lux). Chambers contained an opaque equilateral triangle Plexiglas insert and a staggered grid floor composed of 19 equally spaced stainless steel rods and. Pans underlying the grid floors contained approximately 10 mL of white vinegar solution (Kroger). Vinegar was used to clean chambers before each rat was placed inside. Context C chambers were located in another laboratory suite. The chambers were dimly lit by the chamber house light and contained a flat Plexiglas floor insert. Pans underneath the floors contained approximately 10 mL of Simple Green solution (Sunshine Makers, Inc., Huntington Beach, CA, USA). Odorless sodium hydroxide (5%) was used to clean chambers before each rat was placed inside.

### 2.3. Procedure Experiment 1

Rats underwent fear conditioning during a 40 min training session in Context A. Rats were first allowed to acclimate to the context for three minutes, prior to presentation of the first tone-footshock pairing (30 s, 5 kHz, 75 dB tone co-terminating with a 1 s, 1.5 mA footshock) [15]. Nine additional pairings were presented at a variable interval with an average of four minutes between pairings. One minute after the final tone-shock pairing, the rats were removed from the conditioning box and returned to their homecages.

One day following fear conditioning, all rats were returned to Context A and randomly assigned to extinction or no extinction condition. During each extinction training, 10 tones were presented without footshock in a session that mimicked the tone presentation intervals during training. Rats were removed from the chambers one minute following the termination of the final tone. Extinction training lasted eight days. Rats that did not undergo extinction were placed in the chamber each day for an equivalent 40 min session with no tones or footshocks presented.

One day following the completion of extinction training, all rats underwent a reactivation/renewal session in a novel chamber (Context C). Rats were allowed to acclimate for three minutes before a single tone presentation (identical to that used during training). Rats were removed from the chambers one minute following the termination of the tone and injected with vehicle or midazolam.

One day following the renewal/reactivation session and injection, all rats underwent a test session in a novel context (Context B). Rats were allowed to acclimate for three minutes before three tone presentations (identical to those used during training). Rats were removed from the chambers one minute following the termination of the third tone.

### 2.4. Procedure Experiment 2

The procedure for Experiment 2 was identical to Experiment 1 except that the reactivation session and injections were administered prior to the commencement of extinction training.

### 2.5. Drugs

The benzodiazepine midazolam (MDZ; Midwest Pharmaceuticals, Inc., Farmington Hills, MI, USA), was injected intraperitoneally at a dose of 3 mg/kg diluted in sterile isotonic saline at a volume of 1 mL/kg one minute following tone termination in the reactivation/renewal session [11]. In control rats, the vehicle was intraperitoneally injected at the same volume.

### 2.6. Data Analysis

All statistics were calculated using SPSS version 20.0. Factorial (extinction type and drug injection) and repeated measures (extinction day and tone presentation) analyses of variance (ANOVAs) were conducted to analyze the percentage of time spent freezing during the baseline, tone, and context test periods. A priori planned comparisons between groups were performed using Fisher’s LSD. A critical value α = 0.05 was used for all analyses.

## 3. Results

### 3.1. Experiment 1: Extinction and Renewal Permit Reconsolidation of an Overtrained Fear Memory

**Figure 1:**
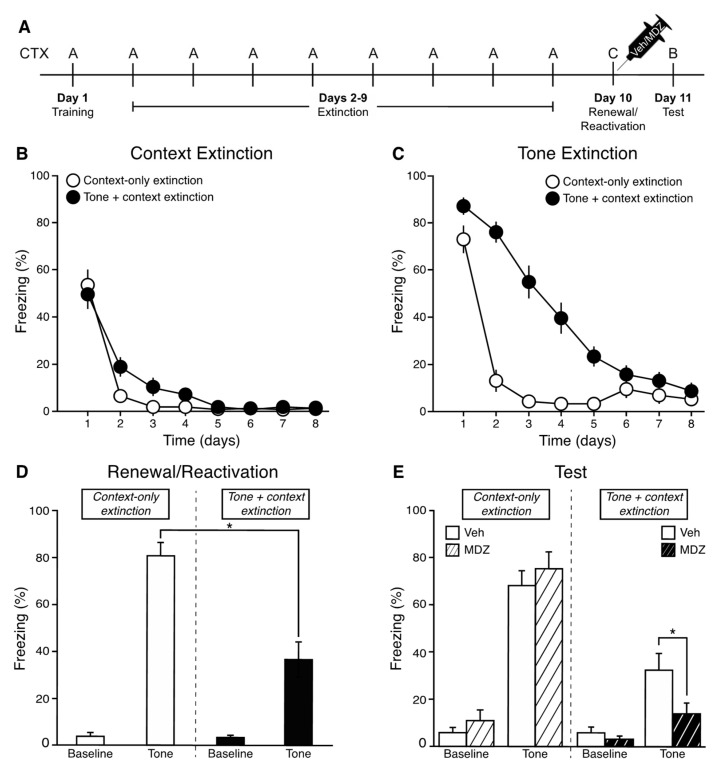
Timeline of Experiment 1 (**A**). Freezing during the first 3 min (context) of each extinction session (**B**). Freezing during the first tone presentation (or equivalent) of each extinction session (**C**). Freezing during the 3 min baseline and tone presentation of the renewal/reactivation session (**D**). Freezing during the 3 min baseline and 3 tone presentations (averaged) of the test (**E**).

Following 10 tone-footshock pairings in Context A, rats underwent context-only or tone + context fear extinction in Context A, followed by a tone renewal/reactivation session in Context C. Systemic injection of vehicle or midazolam occurred immediately following the renewal/reactivation session. The next day, rats were tested for fear of the tone in Context B (see Figure 1A).

#### 3.1.1. Extinction

Freezing during the 3 min baseline period (context) and the 30-s period during the first tone presentation (or equivalent time for context-only extinction animals) were analyzed across each of the 8 days of extinction. As shown in Figure 1B, all rats showed a significant decrease in context freezing during the first 3 min across sessions [*F*(7,231) = 88.72, *p* < 0.001]. There were no main effects of extinction type [*F*(1,33) = 1.75, *p* > 0.05], no differences between future drug injection assignment conditions [*F*(1,33) = 1.36, *p* > 0.05], and no interactions [Ps > 0.05]. For tone + context extinction groups, freezing was assessed during the first tone presentation on each extinction day. For context-only extinction, freezing was assessed during the equivalent 30-s period, although no tone was presented. As shown in Figure 1C, freezing during the tone (or equivalent) significantly decreased in all groups across the 8 days of extinction [*F*(7,231) = 102.75, *p* < 0.001]. In addition, there was a significant main effect of extinction type [*F*(1,33) = 37.93, *p* < 0.001], with tone + context extinction animals showing a gradual decrease in tone freezing over days. Context-only extinction animals show high freezing during the first tone equivalency (context-only) period, but then comparably low levels of freezing during the tone equivalencies on days 2–8. High freezing in the context-only rats on extinction day 1 reflects high context freezing and demonstrates that significant context extinction occurs on day 1 (consistent with Figure 1B). There was no main effect of drug injection [*F*(1,33) = 2.16, *p* > 0.05] and no interactions [Ps > 0.05].

#### 3.1.2. Renewal/Reactivation

One day following the last day of extinction, rats were placed into a novel context and, after 3 min, they received a single tone presentation. As shown in Figure 1D, baseline freezing in the novel context was very low across all groups (<5%) and there were no main effects of extinction type [*F*(1,33) = 0.07, *p* > 0.05], no differences between future drug injection assignment conditions [*F*(1,33) = 0.18, *p* > 0.05], and no extinction type X future drug assignment interaction [*F*(1,33) 0.54, *p* > 0.05]. During the single renewal/reactivation tone, rats that received context-only extinction show significantly higher levels of tone freezing compared to rats that received tone + context extinction [*F*(1,33) = 20.54, *p* < 0.001]. However, there were no differences between future drug injection assignment conditions [*F*(1,33) = 0.54, *p* > 0.05] and no extinction type X future drug assignment interaction [*F*(1,33) = 0.36, *p* > 0.05].

#### 3.1.3. Tone Test

The next day, rats were placed into a novel context and after 3 min received three presentations of the tone, separated as they were during training. As shown in Figure 1E, freezing during the baseline period was low in all groups (<12%), and there were no main effects of extinction type [*F*(1,33) = 1.60, *p* > 0.05] or drug injection [*F*(1,33) = 0.17, *p* > 0.05], and no extinction type X drug injection interaction [*F*(1,33) = 2.38, *p* > 0.05]. Across the three test tones, freezing did not differ [*F*(2,66) = 0.93, *p* > 0.05]; thus, the average of the three test tones was calculated and used in subsequent analyses. There was a significant interaction between extinction type and drug injection [*F*(1,33) = 4.34, *p* < 0.05]. Pairwise comparisons using Fisher’s LSD revealed that tone + context extinction rats injected with midazolam froze significantly less than those injected with vehicle [*p* < 0.05]; context-only extinction rats did not show a difference between injection conditions [*p* > 0.05], revealing that only the tone + context extinction rats underwent disruption of reconsolidation with midazolam. In addition, rats injected with vehicle that received tone + context extinction froze significantly less than vehicle-injected rats that received context-only extinction [*p* < 0.05], demonstrating that renewed tone freezing was lower than non-extinguished tone freezing.

### 3.2. Experiment 2: Extinction does not Unmask Reconsolidation of an Overtrained Fear Memory

**Figure 2:**
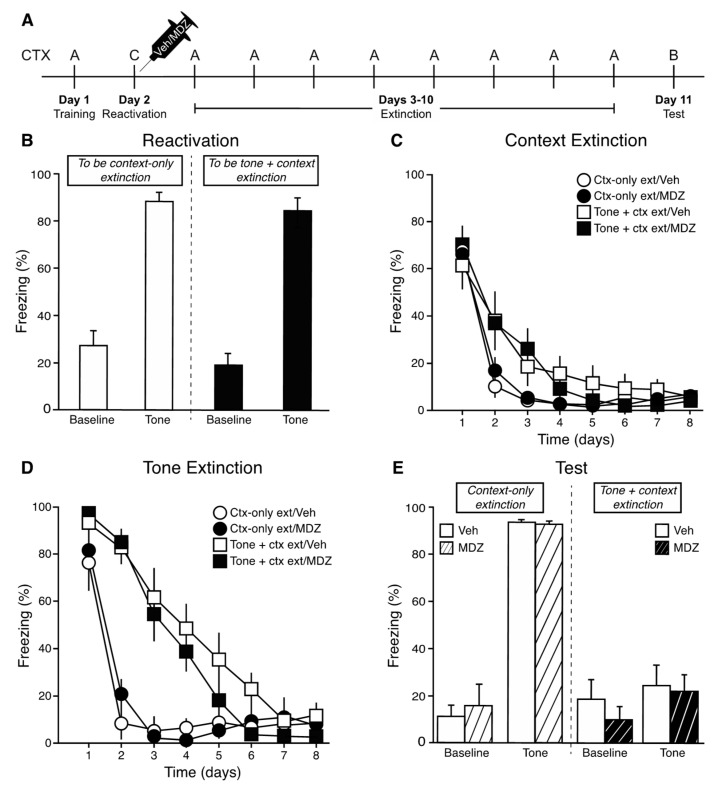
Timeline of Experiment 2 (**A**). Freezing during the 3 min baseline and tone presentation of the reactivation session (**B**). Freezing during the first 3 min (context) of each extinction session (**C**). Freezing during the first tone presentation (or equivalent) of each extinction session (**D**). Freezing during the 3 min baseline and 3 tone presentations (averaged) of the test (**E**).

Following 10 tone-footshock pairings, rats underwent a tone memory reactivation session. Systemic injection of vehicle or midazolam occurred immediately following the reactivation session. Beginning the next day, rats underwent 8 days of context-only or tone + context extinction (see Figure 2A).

#### 3.2.1. Reactivation

One day following acquisition, rats were placed into a novel context (Context C) and after 3 min they received a single tone presentation. Baseline freezing in the novel context was moderate across all groups (15–32%; Figure 2B), showing some generalization between the acquisition and reactivation contexts. However, there were no differences between future extinction type [*F*(1,35) = 1.04, *p* > 0.05] or future drug injection assignment conditions [*F*(1,35) = 1.50, *p* > 0.05], and no future extinction type X future drug assignment interaction [*F*(1,35) = 0.002, *p* > 0.05]. During the single reactivation tone, all groups froze at high levels (85–90%; Figure 2B) and there were no differences between future extinction type [*F*(1,35) = 0.39, *p* > 0.05] or future drug injection assignment conditions [*F*(1,35) = 0.03, *p* > 0.05], and no future extinction type X future drug assignment interaction [*F*(1,35) = 0.03, *p* > 0.05].

#### 3.2.2. Extinction

One day following reactivation, extinction training commenced and continued daily for 8 days. Freezing during the 3 min baseline period (context) and the 30-s period during the first tone presentation (or equivalent time for context-only extinction animals) was analyzed across each of the 8 days of extinction. As shown in Figure 2C, all rats showed a significant decrease in context freezing during the first 3 min across extinction sessions [*F*(7,245) = 92.76, *p* < 0.001]. There were no main effects of extinction type [*F*(1,35) = 3.79, *p* > 0.05] or drug injection [*F*(1,35) = 0.04, *p* > 0.05], and no interactions [*P*s > 0.05]. For tone + context extinction groups, freezing was assessed during the first tone presentation on each extinction day. For context-only extinction, freezing was assessed during the equivalent 30-s period, although no tone was presented. As shown in Figure 2D, freezing during the tone (or equivalent) significantly decreased in all groups across the 8 days of extinction [*F*(7,245) = 88.55, *p* < 0.001]. In addition, there was a significant effect of extinction type [*F*(1,35) = 35.02, *p* < 0.001], with tone + context extinction animals showing a gradual decrease in tone freezing over days. Context-only extinction animals show high freezing during the first tone equivalency, but then comparably low levels of freezing during the tone equivalencies on days 2–8, demonstrating that significant context extinction had occurred on extinction day 1. There was no main effect of drug injection [*F*(1,35) = 0.51, *p* > 0.05] and no interactions [*P*s > 0.05].

#### 3.2.3. Tone Test

The next day, rats were placed into a novel context and after 3 min received three presentations of the tone, separated as they were during training. During the baseline period, all groups froze at low levels (<20%; Figure 2E) and there were no main effects of extinction type [*F*(1,35) = 0.004, *p* > 0.05] or drug injection [*F*(1,35) = 0.11, *p* > 0.05], and no extinction type X drug injection interaction [*F*(1,35) = 0.92, *p* > 0.05]. Across the three test tones, freezing did not differ [*F*(2,70) = 0.15, *p* > 0.05]; thus, the average of the three test tones was calculated and used in subsequent analyses. There was a significant main effect of extinction type [*F*(1,35) = 141.45, *p* < 0.001], with tone + context extinction animals freezing at lower levels compared to context-only extinction animals (Figure 2E). There was no main effect of drug injection [*F*(1,35) = 0.10, *p* > 0.05] and no extinction type X drug injection interaction [*F*(1,35) = 0.02, *p* > 0.05].

## 4. Discussion

We demonstrate that a strong cued fear memory can be rendered susceptible to reconsolidation following cue, but not context, extinction learning. In experiment one, rats that underwent context-only extinction were insensitive to post-reactivation midazolam, showing robust tone fear at test. By contrast, tone + context extinction reduced tone fear, rendering rats susceptible to disruption of reconsolidation. Vehicle-injected tone + context extinction rats display a moderate increase in tone-induced freezing relative to baseline, demonstrating partial renewal of the tone fear memory. However, midazolam-injected tone + context extinction rats show a deficit, indicative of a disrupted reconsolidation process. Taken together, this suggests that extinction learning can weaken the expression of a strong fear memory, rendering a previously immutable memory subject to reconsolidation mechanisms.

An alternate explanation of these results could be a summation process; the combined dampening influence of extinction and reconsolidation manipulations could potentially account for the reduced fear expression. We addressed this possibility by inverting the order of extinction and reactivation, such that reactivation and pharmacological intervention took place prior to extinction training. If the effects observed in Experiment 1 were indeed attributable to a nonspecific summation of reconsolidation and extinction processes, then we would observe a more rapid decrease in freezing across extinction days in tone + context extinction animals that had received midazolam following reactivation, compared to those that received vehicle. Instead, we see identical extinction rates between these groups, suggesting that midazolam had no effect on reconsolidation when the reactivation occurred prior to extinction training.

Mechanistically, our data might be explained by glucocorticoid (GC) secretion during reactivation. GCs are released by the adrenal cortex following activation of the hypothalamic-pituitary-adrenal (HPA) axis. GCs reach their highest concentrations following significant stress exposure, and have been shown to modulate learning and memory, e.g., [29]. For instance, GCs have been shown to enhance memory consolidation while impairing memory retrieval [30,31,32,33]. More recently, GCs have been shown to modulate memory reconsolidation, albeit with mixed results [34]. Stress intensity is an important determinant of GC effects on memory processes, with a number of studies demonstrating an “inverted U-shaped” dose–response curve, e.g., [18,35]. This may explain why reactivation of a strong fear memory (leading to high GC concentrations) may be resistant to reconsolidation, while reactivation of a renewed fear memory (demonstrating moderate fear responding and, likely, moderate GC concentrations) may be sensitive to reconsolidation (and its disruption by amnestic agents such as midazolam). Admittedly, this explanation of our results is speculative at this time, but underscores a need for future experiments aimed at addressing this possibility.

Numerous publications have demonstrated that extended CS reactivation exposure results in behavioral extinction, and subsequent amnestic manipulations attenuate expression of the extinction memory rather than the original association, e.g., [36,37,38,39,40,41,42]. In contrast, Duvarci and colleagues demonstrated that intra-basolateral-amygdalar infusions of the protein synthesis inhibitor, anisomycin, disrupted the reconsolidation of an excitatory CS, regardless of whether the CS reactivation was a single, brief presentation (a conventional “reactivation” session) or presented multiple times or for a long duration (extinction conditions) [43]. Critically, they demonstrate that low fear expression at test is due to disruption of the excitatory CS-US association rather than suppression via a CS-no US extinction memory as vehicle-injected rats showed renewal with a contextual shift, while anisomycin-injected rats did not; thus, the authors conclude extinction is not a barrier to reactivation [43]. Eisenberg et al. [37] suggest that acquisition followed by extinction leads to two competing memory traces (i.e., CS-US and CS-no US) and the memory trace that retains appreciable control over behavior during reactivation may be sensitive to disruption of reconsolidation, whereas the other memory trace is likely to remain stable. This explanation is consistent with our data in experiment 1. Following extinction, renewal allows the initial CS-US memory trace to dominate behavior (i.e., produce freezing) and, thus, it is the CS-US (not the CS-no US) memory trace that undergoes reconsolidation.

Spaced extinction training, as employed in the present work, produces more robust extinction learning relative to massed extinction (i.e., equivalent amount of CS exposure in a single session) that is more resistant to return via mechanisms such as renewal, e.g., [44]. This represents an important distinction between the present work and prior demonstrations of manipulations following massed extinction learning attenuating extinction expression [36,39,40,41,42]; but see [37,38,42].

It is curious that in Experiment 2, rats that had undergone tone + context extinction failed to demonstrate significant renewal of the tone fear memory during test (Figure 2E). Modest renewal effects are not altogether surprising as we employed an AAB renewal design, which results in weaker renewal than other paradigms, e.g., [45]. In addition, the reactivation/renewal session takes place in a third context, effectively resulting in extinction in multiple contexts—an approach that is also known to strengthen extinction learning and attenuate renewal, e.g., [46,47]; but see [48]. Indeed, retrieval-based models that predict retrieval practiced under varied conditions facilitates subsequent retrieval, e.g., [49,50]. Extinction can be understood as a new, “safe” memory that competes for expression with the original, “unsafe” memory; it follows that a more strongly encoded “safe” memory would be more difficult to overcome. It is possible that undergoing a reactivation session prior to extinction training attenuated renewal at test due to a richer encoding process, e.g., [51]. We did not observe this effect when conditions were reversed in experiment 1; this could be because sufficient learning had *already taken place* prior to the “reactivation” session. Thus, another instance of tone-safety might have less relevance to an animal in this condition compared to an animal encountering the tone-safety pairing in a novel context prior to any extinction learning per se.

To some degree, the importance of the order of retrieval and extinction sessions has been explored in the context of the retrieval-extinction procedure [7]. Extinction carried out immediately following a reactivation session produces robust erasure of fear that is insensitive to return via mechanisms such as renewal and spontaneous recovery, e.g., [7,8,52]. However, replication of this phenomenon has been inconsistent, e.g., [53,54], and recent work suggests it may be best explained as a strengthening of extinction learning rather than reconsolidation per se [55]. Notably, so long as both the reactivation and extinction sessions take place within a 6 h window, their order is unimportant [56,57]. This suggests that rich encoding via retrieval practice strengthening the extinction learning (potentially via reconsolidation mechanisms) is the driving force behind this phenomenon [57]. Similar mechanisms may be at play in our renewal data; the renewal practice may be enhancing the formation of the extinction memory in Experiment 2, preventing renewal. By contrast, the presence of a renewal effect in Experiment 1 suggests that, in the case of spaced extinction taking place on the order of days, the session order matters. Importantly, the present work departs significantly from these studies in that extinction sessions take place over the course of eight daily sessions rather than within a six-hour window. Further research is necessary to test these predictions.

## 5. Conclusions

The present experiments demonstrate that a strong fear memory, which is typically resistant to disruption of reconsolidation, can be made susceptible to reconsolidation if expression of the fear memory is weakened at the time of reactivation. Future studies should address whether a similar approach might prove useful in the reconsolidation of strong appetitive memories (e.g., drug-paired). The present data have important translational significance. Circumventing the apparent boundaries on reconsolidation encountered with strong emotional memories could prove invaluable in the treatment of debilitating conditions such as posttraumatic stress disorder and substance use disorder.

## Data Availability

Upon publication of the manuscript, the data presented in this paper will be openly available in Scholarly Commons at http://hdl.handle.net/2374.MIA/6742 accessed ib 30 July 2021.
